# X-box promoter motif searches: from *C. elegans *to humans to novel candidate ciliopathies

**DOI:** 10.1186/2046-2530-4-S1-P56

**Published:** 2015-07-13

**Authors:** G Lauter, K Tammimies, A Bieder, R Torchet, H Matsson, M Peyrard, J Burghoorn, E Castren, J Kere, I Tapia-Páez, P Swoboda

**Affiliations:** 1 Department of Biosciences and Nutrition, Karolinska Institute, Huddinge, Sweden; 2 Neuroscience Center, University of Helsinki, Helsinki, Finland

## Objective

Ciliary defects are known to cause severe genetic disorders, collectively called ciliopathies. We attempt to identify genes involved in human ciliopathies by making use of the evolutionarily conserved X-box promoter motif recognized by ciliogenic RFX transcription factors.

## Methods

We use bioinformatics tools to identify potential RFX target genes across species. We currently focus on genes associated with human dyslexia, the most common learning disorder. In human cell culture systems we test the functionality of X-box motifs, determine target gene expression levels in dependence to RFX and cilia development, and visualize their subcellular protein localization by immunocytochemistry.

## Results

Following the methodology of X-box searches developed in *C. elegans*, we have identified many X-box containing genes in the human genome. Luciferase gene reporter assays show that the dyslexia candidate genes DYX1C1, KIAA0319 and DCDC2 possess a functional X-box motif. Analogous to expression profiles of known cilia markers, DYX1C1, KIAA0319 and DCDC2 gene expression increases during the first 24 h after induction of ciliogenesis. Furthermore, expression levels of DYX1C1, KIAA0319 and DCDC2 change in response to siRNA-mediated knockdown of RFX factors. In line with a proposed ciliary function, the proteins of DYX1C1 and DCDC2 localize at the cilium.

## Conclusions

The evolutionarily conserved X-box promoter motif can be used to identify potential ciliary genes across species. Human dyslexia candidate genes DYX1C1, KIAA0319 and DCDC2 possess a functional X-box motif and are regulated by RFX factors. Human cell lines thus provide an easily accessible and rapid way to test X-box functionality.

